# Nonlinear dose–response effects of exercise interventions on post-stroke depression: a systematic review and meta-analysis

**DOI:** 10.3389/fpubh.2026.1876753

**Published:** 2026-07-14

**Authors:** Meiyao Liu, Ziqi Gao, Yaxin Wang, Huimin Yang, Chang Liu, Jiahe Wu, Shenghui Liang, Shiyue Shao, Ziheng Zhang, Jianxin Kang, Xin Liu

**Affiliations:** Graduate School, Harbin Sport University, Harbin, China

**Keywords:** dose–response relationship, exercise intervention, meta-analysis, metabolic equivalent, post-stroke depression

## Abstract

**Objective:**

To systematically evaluate the effects of exercise interventions on post-stroke depression (PSD) and to clarify the dose–response relationship between physical activity dosage and depressive symptoms in individuals with PSD.

**Methods:**

A structured and comprehensive search was conducted in PubMed, Web of Science, Embase, Scopus, and the Cochrane Library. Restricted cubic spline models were applied to examine the dose–response association between physical activity dosage and depressive symptoms in PSD.

**Results:**

A total of 27 publications comprising 31 randomized controlled trials were included, involving 2,247 participants. The meta-analysis indicated that exercise intervention was associated with a modest improvement in depressive symptoms among patients with PSD [SMD = −0.15, 95% CI (−0.23, −0.07), *p* < 0.01], with moderate heterogeneity (*I*^2^ = 41.9%). Dose–response analysis revealed a non-linear association between exercise dosage and symptom improvement, with the greatest apparent benefit observed at approximately 801 MET-min/week. Subgroup analyses suggested that more favorable improvements were more commonly observed in interventions characterized by resistance training [SMD = −0.55, 95% CI (−0.85, −0.25)], a frequency of 1–2 sessions per week [SMD = −0.41, 95% CI (−0.67, −0.15)], a session duration of ≤30 min [SMD = −0.44, 95% CI (−0.70, −0.19)], and an intervention duration of 9–12 weeks [SMD = −0.16, 95% CI (−0.27, −0.05)].

**Conclusion:**

Moderate-dose exercise intervention, approximately 801 MET-min/week, was associated with a modest improvement in post-stroke depressive symptoms. These findings provide a reference for optimizing exercise dosage and informing individualized prescription strategies for patients with PSD.

**Systematic review registration:**

https://www.crd.york.ac.uk/PROSPERO/view/CRD420251167322, identifier (CRD420251167322).

## Highlights

- Exercise interventions were associated with a modest improvement in post-stroke depressive symptoms.
- A non-linear relationship was observed between weekly exercise dose and symptom improvement.
- A weekly exercise volume of around 801 MET-min/week was associated with the greatest model-predicted benefit.
- These findings may help inform individualized exercise prescription for post-stroke depression, but should be interpreted with caution.

## Introduction

Stroke remains the second leading cause of death worldwide and a major contributor to long-term disability, exerting enduring effects on physical function, cognitive performance, and social participation (1). Post-stroke depression (PSD) is a prevalent yet frequently overlooked neuropsychiatric sequela, typically characterized by persistent low mood, anhedonia, fatigue, and sleep disturbance (2, 3). Its pathogenesis is multifactorial, reflecting the interplay among structural brain injury, biological stress responses, and maladaptive processes of illness adjustment (4, 5).

According to data from the World Health Organization (WHO), approximately 150 million stroke survivors worldwide are at risk of depression; nearly one-third develop depressive symptoms within the first year after stroke, and the cumulative prevalence reaches 31% within 5 years (6, 7). Against the backdrop of improving survival and alleviating the long-term functional and psychological burden associated with stroke, PSD has emerged as an important focus in both rehabilitation medicine and public health (8). Importantly, PSD may undermine patients’ motivation for rehabilitation and treatment adherence, while further exacerbating functional decline through its adverse effects on cognitive processing, social participation, and self-efficacy (8, 9). Accordingly, identifying effective intervention strategies for PSD is of clear clinical importance.

In recent years, although antidepressants have remained a cornerstone of PSD treatment, their clinical utility has been constrained by uncertain efficacy, potential adverse effects, and the risk of withdrawal after discontinuation, underscoring the need for non-pharmacological alternatives (10, 11). Exercise intervention has attracted increasing attention as a promising adjunctive strategy in PSD management because of its high feasibility, relatively controllable risk profile, and ease of integration into routine rehabilitation programs (12, 13). Mechanistically, exercise may exert antidepressant effects by modulating key neurotransmitter systems, regulating hypothalamic–pituitary–adrenal (HPA) axis activity, and upregulating brain-derived neurotrophic factor (BDNF), thereby fostering a neurogenic milieu and mitigating neurotoxicity-related damage (14, 15). Collectively, these neurobiological adaptations may contribute to the alleviation of depressive symptoms.

A growing body of randomized controlled trials (RCTs) and systematic reviews suggests that exercise intervention holds therapeutic potential for reducing post-stroke depressive symptoms. Nevertheless, the current literature remains substantially limited by small sample sizes and the lack of comprehensive comparative analyses across different intervention protocols. The most effective exercise modalities and the underlying dose–response relationship have yet to be clearly established.

Accordingly, the present study conducted a systematic review and meta-analysis to quantify the overall effects of exercise interventions on PSD. Beyond deriving pooled effect estimates, we further performed a dose–response analysis to examine the associations of key prescription components, including intervention duration, frequency, and session length, with depressive outcomes. By additionally exploring between-study heterogeneity and its potential sources, we sought to provide an evidence-based reference for the individualized optimization of exercise prescriptions and to offer further insight into rehabilitation strategies for patients with post-stroke depression.

## Methods

This systematic review and meta-analysis was conducted in strict accordance with the Preferred Reporting Items for Systematic Reviews and Meta-Analyses (PRISMA) guideline (16). The study protocol was prospectively registered in PROSPERO under registration number CRD420251167322.

### Search strategy

A systematic literature search was performed in PubMed, Embase, Scopus, Web of Science, and the Cochrane Library from database inception to February 16, 2026. Eligibility was restricted to English-language publications and RCTs. The search strategy was developed using a combination of Medical Subject Headings (MeSH) and free-text terms. Four core concepts were incorporated into the search framework: “stroke,” “depression,” “exercise intervention,” and “randomized controlled trial.” The search syntax was adapted to the indexing rules and retrieval requirements of each database. The full search strategy is provided in Supplementary material A.

### Eligibility criteria

Studies were considered eligible if they met the following criteria. Participants were required to be stroke survivors with post-stroke depression or clinically relevant depressive symptoms, as identified by clinical evaluation or validated psychometric instruments. Interventions comprised structured and regular exercise programs, such as aerobic training, resistance training, mind–body exercise, or multimodal exercise. In the control group, participants did not receive any structured exercise intervention. In addition, studies were required to report at least one quantitative outcome related to post-stroke depression, assessed using standardized depression rating scales, such as the Hamilton Depression Rating Scale (HAMD), Beck Depression Inventory (BDI), or Patient Health Questionnaire-9 (PHQ-9).

Studies were excluded if they met any of the following criteria. Animal experiments and other non-human studies were excluded. Duplicate publications were screened, and when duplicate reports were identified, the version with the larger sample size or more comprehensive data was retained. Studies were also excluded if the full text was unavailable or if relevant data on depressive outcomes could not be extracted. In addition, studies that did not assess PSD using standardized depression measures were not considered eligible. Other study designs, including narrative reviews, systematic reviews, meta-analyses, conference abstracts, and commentaries, were also excluded. Finally, studies involving participants with severe psychiatric or neurological comorbidities that could affect depressive outcomes or exercise responses were excluded, including schizophrenia, bipolar disorder, dementia, severe cognitive impairment, Parkinson’s disease, multiple sclerosis, epilepsy, brain tumors, and severe traumatic brain injury.

### Study selection

All retrieved records were imported into EndNote (EndNote 21, Clarivate Analytics, Philadelphia, PA, USA) for reference management, and duplicate entries were removed prior to screening. Eligible studies were identified through a 2-stage process. First, titles and abstracts were screened against the predefined inclusion and exclusion criteria. Full texts of potentially relevant articles were then retrieved and examined in detail to determine final eligibility. Any disagreements were resolved through discussion and consensus, with consultation from a third researcher when necessary.

Multi-arm RCTs were included when they met the eligibility criteria. When a single study contained multiple intervention arms, such as different exercise modalities, intensities, or intervention durations, and all were relevant to the study objective, each eligible intervention arm was treated as an independent comparison. Where multiple intervention arms shared a common control group, the sample size of the control group was appropriately divided, or the intervention groups were combined as appropriate, to avoid double counting and reduce the risk of unit-of-analysis errors.

### Data extraction

Data extraction and tabulation were performed using Microsoft Word and Excel (Windows version 2,601, Build 19,628, Microsoft Corporation, Redmond, WA, USA). The following information was extracted: (1) general study characteristics, including first author, year of publication, and journal; (2) baseline characteristics of the intervention and control groups, including sample size, mean age, intervention duration, session length, and exercise frequency; and (3) outcome measures, particularly quantitative indicators related to post-stroke depression. When required data were incomplete or unclear, attempts were made to contact the corresponding authors to obtain additional information.

To synthesize intervention effects across studies, treatment effects were evaluated by calculating changes in depressive scale scores on the basis of the mean values, standard deviations, and sample sizes reported before and after the intervention in each study group:


$${\mathrm{MD}}_{\text{diff}}=M_{\text{post}}-M_{\mathrm{pre}}$$

Where *MD_diff_* denotes the raw mean difference, *Mpost* represents the reported post-intervention mean, and *Mpre* represents the reported pre-intervention mean.

Standardized Mean Difference (SMD) was calculated as follows:


$$\mathrm{SMD}=\frac{M_{1}-M_{2}}{{\mathrm{SD}}_{\text{pooled}}}$$

$${\mathrm{SD}}_{\text{pooled}}=\sqrt{\frac{(n_{1}-1){\mathrm{SD}}_{1}^{2}+(n_{2}-1){\mathrm{SD}}_{2}^{2}}{n_{1}+n_{2}-2}}$$

Where M_1_ and M_2_ represent the mean values of the intervention and control groups, respectively, and SDpooled represents the pooled standard deviation of the two groups.

The standard deviation of the change score (SD_diff_) was calculated as follows:


$${\mathrm{SD}}_{\text{diff}}=\sqrt{SD_{\mathrm{pre}}^{2}+SD_{\text{post}}^{2}-2r\times SD_{\mathrm{pre}}\times SD_{\text{post}}}$$

Where SDpre and SDpost represent the standard deviations before and after the intervention, respectively, and r represents the correlation coefficient between pre-intervention and post-intervention measurements. When the required information was unavailable, a correlation coefficient of 0.5 was assumed for the calculation of SDdiff (17).

To combine subgroups, the following approach was applied. Assuming that subgroup A had a sample size of *N*_1_, mean of *M*_1_, and standard deviation of SD_1_, and subgroup B had a sample size of *N*_2_, mean of *M*_2_, and standard deviation of SD_2_, the combined sample size was calculated as *N* = *N*_1_ + *N*_2_, and the combined mean was calculated as *M* = (*N*_1_*M*_1_ *+ N*_2_*M_2_*)/(*N*_1_ *+ N*_2_). The combined standard deviation was calculated as follows:


$$\mathrm{SD}=\sqrt{\frac{\left(N_{1}-1)SD_{1}^{2}+(N_{2}-1)SD_{2}^{2}+\frac{N_{1}N_{2}}{N_{1}+N_{2}}(M_{1}^{2}+M_{2}^{2}-2M_{1}M_{2}\right)}{N_{1}+N_{2}-1}}$$

If data from 3 or more subgroups required pooling, the same formula was applied sequentially; that is, by first combining 2 subgroups and then combining the resulting data with the third subgroup, and so forth.

Given that most included studies had relatively small sample sizes, Hedges’ g was adopted as the standardized effect size in the dose–response model to reduce small-sample bias. Hedges’ *g* was derived by applying a small-sample correction to the SMD, using the following formula:


$${\text{Hedges}}^{'}\;g=\mathrm{SMD}\times (1-\frac{3}{4(n_{1}+n_{2})-9})$$

Where *n_1_* and *n*_2_ denote the sample sizes of the intervention and control groups, respectively (18).

### Extraction of physical activity intervention dose

To evaluate the dose–response relationship between physical activity intervention and changes in post-stroke depressive symptoms, physical activity exposure was converted into metabolic equivalent minutes per week (MET-min/week) based on the metabolic equivalent (MET) framework (19). Restricted cubic spline models were then applied to characterize potential non-linear dose–response associations. The exercise dose analyzed in this study reflects the prescribed intervention dose rather than the actual completed dose, as calculations were derived from the exercise modality, intensity, session duration, and training frequency reported in the intervention protocols of the included studies. For each study, the median or mean level of physical activity intervention was assigned to the corresponding effect size for post-stroke depression outcomes. When these data were unavailable, the midpoint of the upper and lower boundaries of each category was used as the representative value.

Dose–response analyses were performed using restricted cubic spline (RCS) models to explore potential nonlinear associations between exercise dose and changes in depressive symptoms. Exercise dose was standardized as MET·min/week by integrating exercise intensity, frequency, and duration across studies. Following current methodological recommendations, four knots were placed at the 5th, 35th, 65th, and 95th percentiles of the dose distribution to provide sufficient flexibility while minimizing the risk of overfitting (20, 21). Nonlinearity was assessed by testing whether the coefficients of the spline transformations were jointly equal to zero. The dose associated with the greatest improvement in depressive symptoms was identified from the fitted dose–response curve. Graphical presentation and model estimation procedures followed established approaches for flexible dose–response modeling (22). All dose–response analyses were conducted using R software (R Foundation for Statistical Computing, Vienna, Austria).

### Quality assessment and risk of bias

The risk of bias in RCTs was assessed using the Cochrane Risk of Bias tool, version 2 (RoB 2; Cochrane Collaboration, London, UK). RoB 2 evaluates bias across 5 key domains: the randomization process, deviations from intended interventions, missing outcome data, measurement of the outcome, and selection of the reported result. Each domain was judged as “low risk of bias,” “some concerns,” or “high risk of bias.” Assessments were conducted independently by 2 researchers, and any disagreements were resolved through discussion until consensus was reached. Publication bias was evaluated through visual inspection of funnel plot asymmetry and Egger’s test. These assessments informed the subsequent sensitivity analyses and aided in the interpretation of between-study heterogeneity.

### Statistical analysis

All statistical analyses were performed using Stata (Stata 18.0, StataCorp LLC, College Station, TX, USA). As the outcomes were continuous variables, weighted mean differences (WMDs) were calculated when the same measurement instrument was used across studies, whereas standardized mean differences (SMDs) were applied when outcomes were assessed using different scales. Pooled estimates were reported with 95% confidence intervals (CIs).

Heterogeneity was assessed using the *I*^2^ statistic and the corresponding *p* value. When *I*^2^ < 50% and *p* > 0.1, between-study heterogeneity was considered low, and a fixed-effect model was applied. When *I*^2^ ≥ 50% and *p* < 0.1, heterogeneity was considered substantial, and a random-effects model was used. Model selection also considered the clinical and methodological characteristics of the included studies (23). Sensitivity analyses were performed to assess the robustness and stability of the pooled results. Subgroup analyses were further conducted to explore potential sources of heterogeneity. Statistical significance was defined as *p* < 0.05. Sensitivity analyses were performed using a leave-one-out approach, in which each study was sequentially removed and the meta-analysis was repeated to assess the influence of individual studies on the pooled effect estimates and the robustness of the overall findings.

## Results

### Study selection

**Figure 1:**
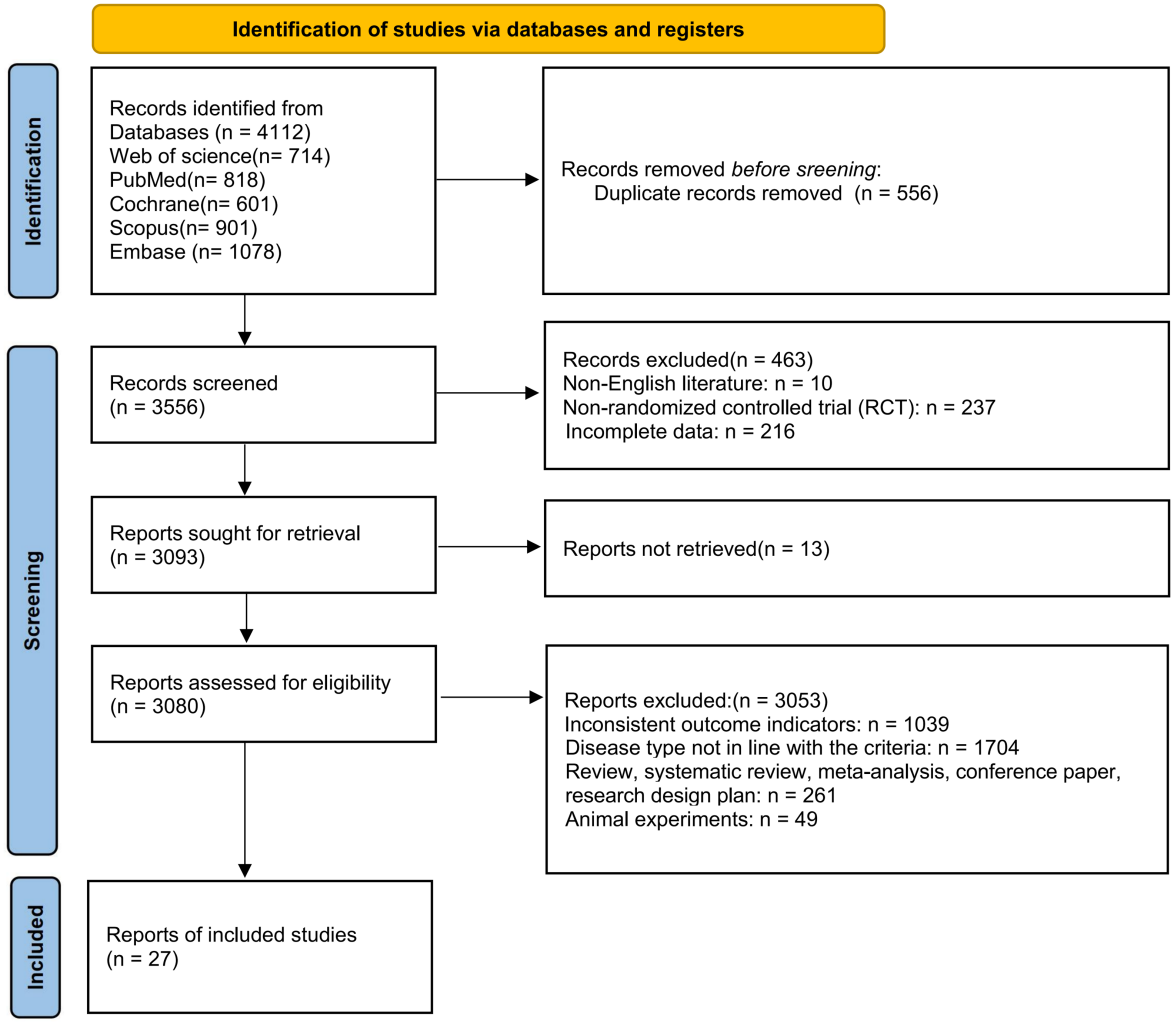
PRISMA flow diagram of study selection.

A total of 4,112 records were identified through a systematic search of PubMed, Embase, Scopus, Web of Science, and the Cochrane Library. The detailed study selection process is presented in Figure 1. After removal of duplicate records, screening of titles and abstracts, and exclusion of ineligible articles, 27 publications were ultimately included (24–50), comprising 31 RCTs.

### Characteristics of the included studies

A total of 27 publications comprising 31 RCTs were included, involving 2,247 patients with post-stroke depression, of whom 1,134 were assigned to the intervention group and 1,113 to the control group. All participants were adults, with a reported mean age of 61.6 ± 10.9 years. With respect to the interventions, participants in the experimental groups received structured exercise programs of varying modalities, including aerobic training, resistance training, multimodal exercise, and mind–body exercise. Control groups generally received usual care or no structured exercise intervention.

Exercise prescriptions were characterized by 3 principal parameters: intervention duration, session length, and exercise frequency. Intervention duration ranged from 1 to 18 weeks, session length varied from 30 to 120 min, and exercise frequency ranged from 1 to 14 sessions per week. Depressive outcomes were assessed using validated standardized instruments, including the HAMD, BDI, PHQ-9, HADS-D, GDS, CES-D, and PROMIS Depression. These instruments were used across the included studies to assess depressive symptoms in post-stroke populations. Across all scales, lower scores indicate less severe depressive symptoms (Table 1).

**Table 1:** Basic characteristics of included studies (*n* = 31).

First author	Country	Experimental group	Control group	Age (years)	Exercise duration (weeks)	Frequency (sessions/week)	Intensity (MET)	Time per week (mins)	Type	METs_min_week	Outcome Indicator
Ademoyegun et al. (24)	Nigeria	11	13	64.2 ± 9.36	1	7	3.3	45	Multimodal	1039.5	HADS
Aguiar et al. (25)	Canada	14	19	52 ± 11	12	3	7.3	40	Aerobic training	876	PHQ-9
Aidar et al. (27)	Brazil	15	15	50.3 ± 9.1	12	2	5.5	50	Aerobic training	550	BDI
Aidar et al. (27)	Brazil	44	49	51.7 ± 8.0	12	3	5.5	60	Resistance training	990	BDI
Aidar et al. (28)	Brazil	15	12	52 ± 11	12	2	5.5	50	Aerobic training	550	BDI
Chan et al. (29)	Australia	19	17	67.1 ± 15.4	6	1	5.5	90	Mind–body exercise	495	HRSD
Faulkner et al. (30)	New Zealand	13	12	69 ± 11	8	2	5.5	90	Resistance training	990	HADS
Gjellesvik et al. (31)	Norway	16	16	57.6 ± 9.2	8	3	7	35	Aerobic training	735	HADS
Holmgren et al. (32)	Sweden	8	6	77.7 ± 7.6	5	3	5	100	Multimodal	1,500	GDS
Jun et al. (35)	South Korea	149	157	55.10 ± 17.23	8	3	2	60	Aerobic training	360	CES-D
Lai et al. (36)	USA	50	50	68.5 ± 9.0	12	3	3.8	60	Multimodal	684	GDS
Lapointe et al. (37)	Canada	24	24	69.2 ± 10.7	18	3	8.8	40	Aerobic training	1,056	HADS
Lapointe et al. (37)	Canada	11	11	69.2 ± 10.7	18	3	5.8	40	Aerobic training	696	HADS
Lenoir Dit Caron et al. (38)	France	11	11	67.6 ± 11.1	12	3	2.3	60	Multimodal	414	BDI
Ihle-Hansen et al. (33)	Norway	15	13	71.4 ± 11.3	18	7	3	49	Multimodal	1,029	HADS
Liu et al. (39)	China	51	51	57.59 ± 11.46	8	14	2.3	30	Mind–body exercise	966	HAMD
Immink et al. (34)	Australia	93	93	56.1 ± 13.6	10	7	2.3	47	Mind–body exercise	756.7	GDS
Maček et al. (40)	Croatia	27	28	68.20 ± 6.80	3	3	5.5	45	Functional	742.5	HADS
Mayo et al. (41)	Canada	21	20	61 ± 12	12	2	5.5	45	Aerobic training	495	S-GDS
Mulder et al. (42)	Netherlands	21	20	61.3 ± 11.5	8	5	3.8	30	Multimodal	570	HADS
Mulder et al. (42)	Netherlands	23	23	61.3 ± 11.5	8	5	3.8	30	Multimodal	570	PROMIS
Nindorera et al. (43)	Burundi	125	117	50.9 ± 10.7	12	3	3	120	Functional	1,080	HADS
van de Port et al. (48)	Netherlands	16	8	56 ± 10	12	2	5.5	90	Functional	990	HADS
Rosenfeldt et al. (44)	USA	16	8	51 ± 12	8	3	6.8	45	Aerobic training	918	CES-D
Rosenfeldt et al. (44)	USA	23	21	51 ± 12	8	3	6.8	45	Aerobic training	918	CES-D
Sims et al. (45)	Australia	30	30	67.13 ± 15.23	10	2	3.5	60	Resistance training	420	CES-D
Sun et al. (46)	China	32	28	65.23 ± 6.29	3	7	3.3	60	Mind–body exercise	1,386	HAMD
Taylor-Piliae et al. (47)	USA	53	48	69.9 ± 10.0	12	3	6	60	Mind–body exercise	1,080	CES-D
Taylor-Piliae et al. (47)	USA	44	48	69.9 ± 10.0	12	3	5	60	Functional	900	CES-D
Vloothuis et al. (49)	Netherlands	31	27	59.26 ± 15.01	8	5	3.8	30	Functional	570	HADS
Xie et al. (50)	China	113	118	60.9 ± 8.7	12	5	3.3	60	Mind–body exercise	990	BDI

HADS, hospital anxiety and depression scale; PHQ, patient health questionnaire; BDI, beck depression inventory; HAMD, hamilton depression rating scale; GDS, geriatric depression scale; CES-D, center for epidemiologic studies depression scale; S-GDS, short-form geriatric depression scale; PROMIS, patient-reported outcomes measurement information system.

### Quality assessment

Methodological quality was evaluated using the RoB 2. Most trials were judged to be at low risk of bias in the domains of the randomization process, missing outcome data, and selective reporting. In the domain of deviations from intended interventions, several studies were rated as having some concerns, primarily owing to insufficient detail regarding intervention implementation and related reporting. Some studies also raised some concerns in the measurement of outcomes domain. In addition, a small number of trials were judged to be at high risk of bias with respect to missing outcome data or other sources of bias. Overall, the included studies were considered to have an acceptable level of risk of bias (Supplementary material B).

### Meta-analysis results

#### Effects of exercise intervention on post-stroke depression

**Figure 2:**
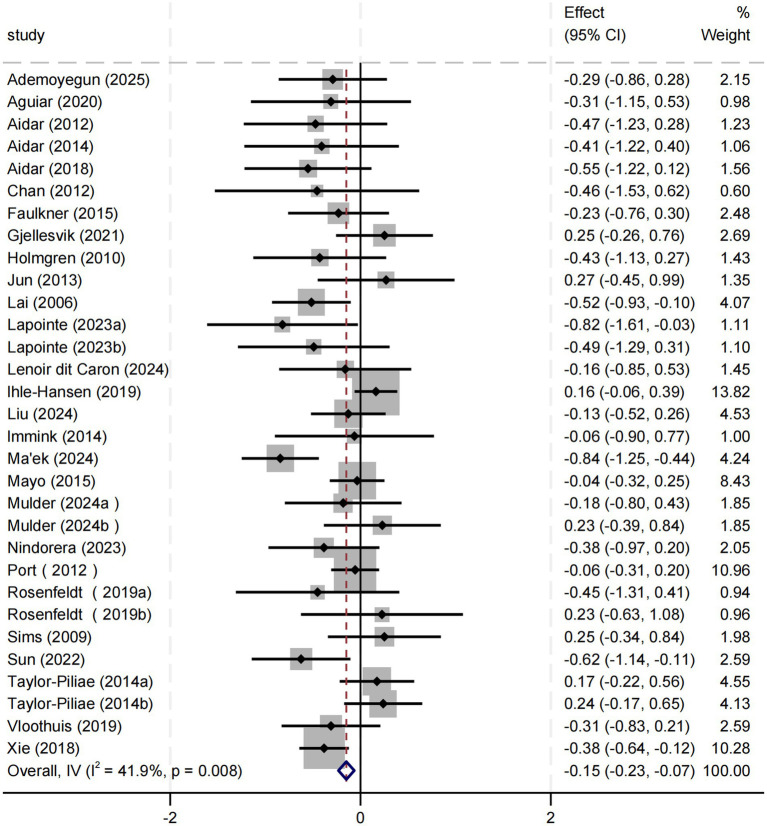
Forest plot illustrating the pooled effects of exercise interventions on post-stroke depression.

A total of 27 publications comprising 31 RCTs were included to evaluate the effects of exercise interventions on post-stroke depressive symptoms (Figure 2). Between-study heterogeneity was moderate [*I*^2^ = 41.9%, *p* = 0.008], and a fixed-effect model was therefore used to pool the effect sizes. The meta-analysis showed that, compared with the control group, exercise intervention was associated with a modest reduction in the severity of depressive symptoms [SMD = −0.15, 95% CI (−0.23, −0.07), *p* < 0.01]. To further evaluate the potential impact of between-study heterogeneity, a 95% prediction interval was calculated using a random-effects model. The pooled effect estimate was [SMD = −0.18, 95% CI (−0.31, −0.06)], and the corresponding 95% prediction interval ranged from −0.649 to 0.276.

#### Publication bias

**Figure 3:**
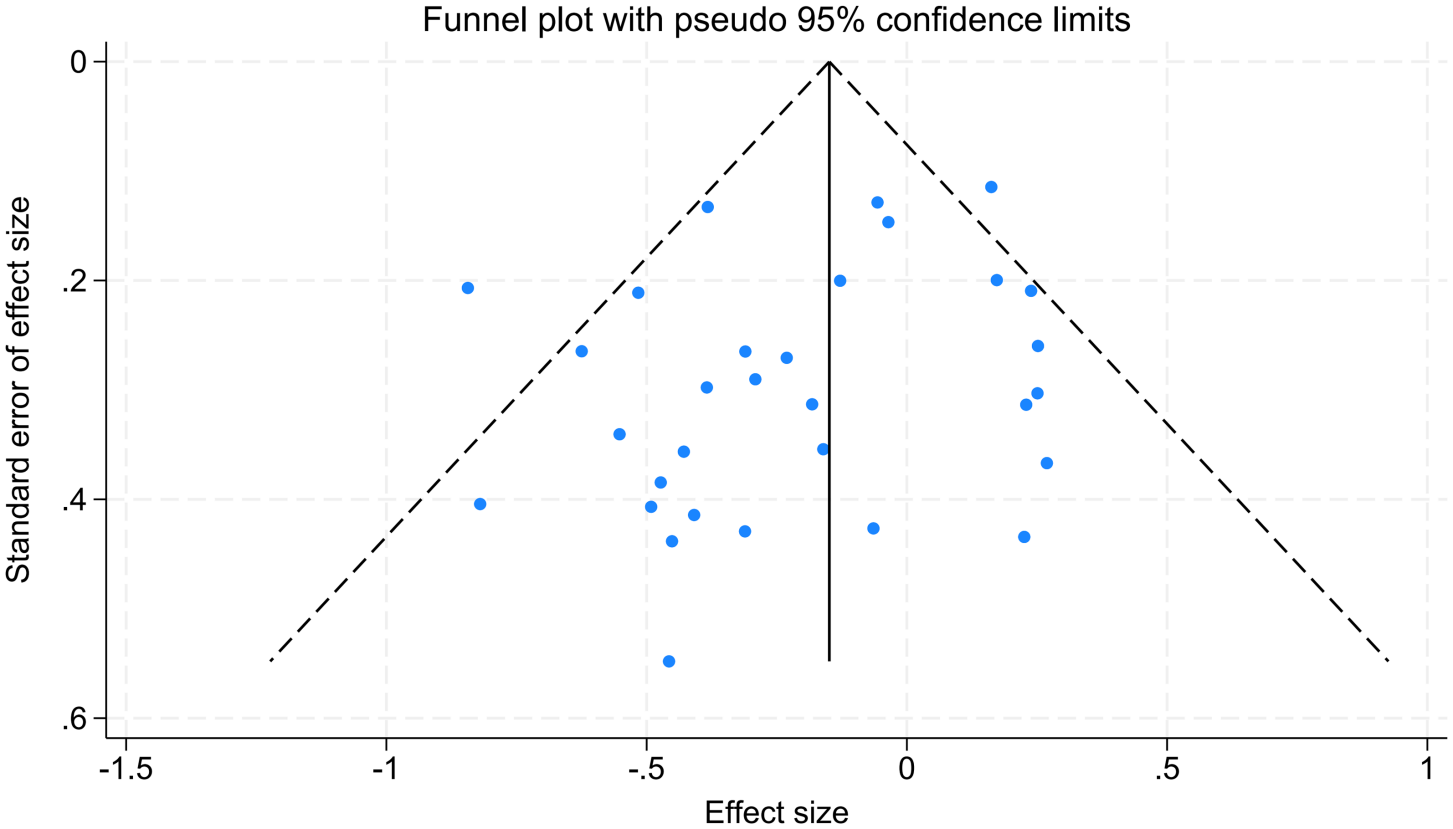
Funnel plot assessing publication bias for the effects of exercise on post-stroke depression.

Publication bias was preliminarily assessed using a funnel plot of the included studies (Figure 3). Most study points were distributed on both sides of the pooled effect size, and the overall pattern appeared broadly symmetrical, with progressive narrowing toward the top of the plot. In addition, Egger’s test did not indicate significant publication bias [*p* = 0.137]. Taken together, these findings suggest a low risk of publication bias in the present meta-analysis.

#### Dose–response analysis

**Figure 4:**
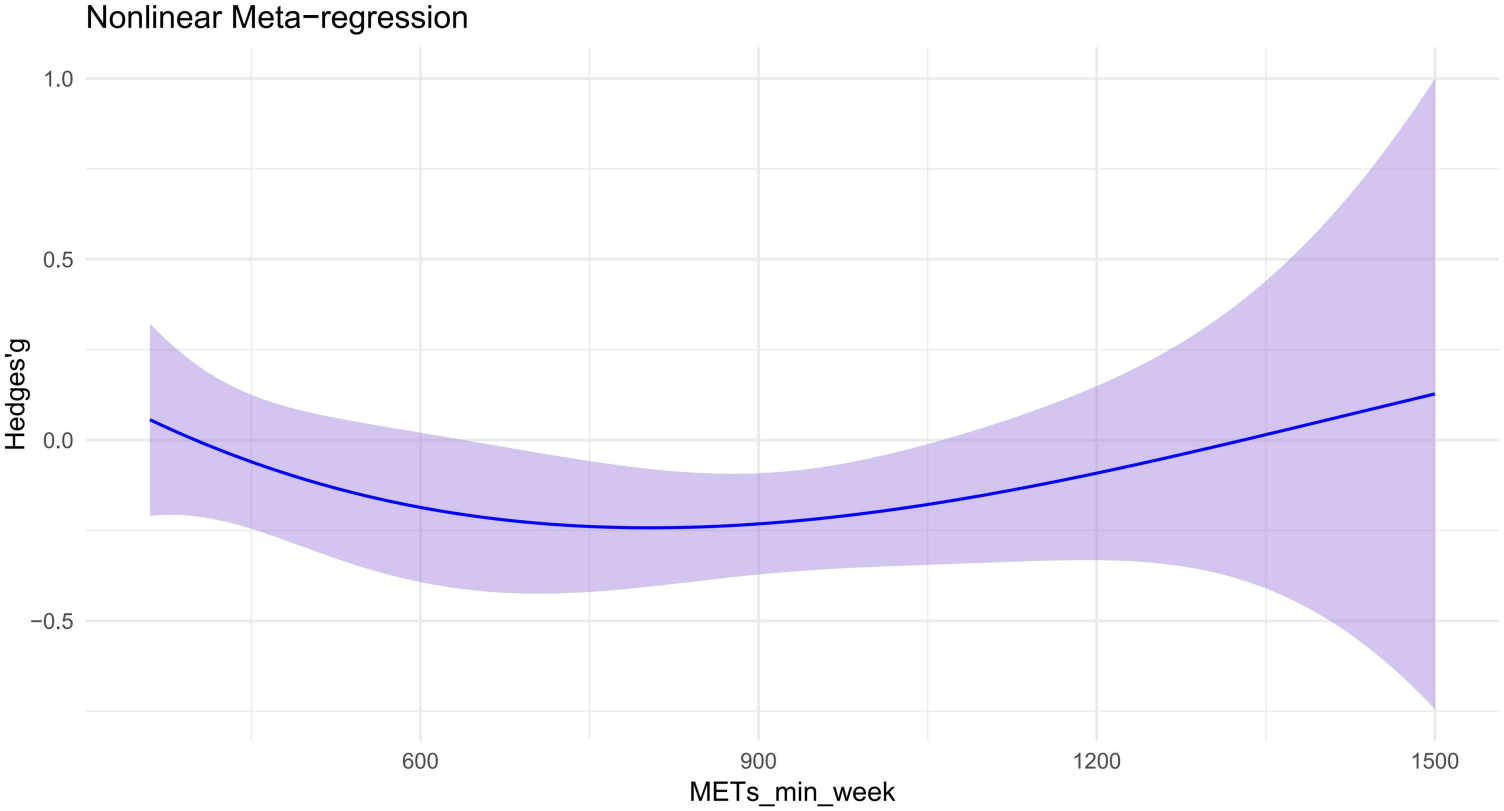
Nonlinear dose–response association between exercise volume and post-stroke depression.

The results demonstrated a significant non-linear dose–response relationship between exercise dosage and the magnitude of improvement in post-stroke depression. The test for nonlinearity was statistically significant (*p* < 0.001). The corresponding dose–response curve is presented in Figure 4. To enhance the stability of dose–response modeling under small-sample conditions, Hedges’ g was used as the effect size metric in the non-linear meta-regression analysis. Overall, the dose–response curve exhibited a clear U-shaped non-linear pattern. When the exercise dose reached approximately 801.11 MET-min/week, the model-predicted antidepressant effect approached its relative peak, as reflected by the lowest Hedges’ g value, indicating the greatest expected improvement in depressive symptoms.

Within the lower-dose range, approximately 300–500 MET-min/week, the effect size gradually declined as exercise dosage increased, suggesting a progressive strengthening of the antidepressant effect. When the dose ranged from 600 to 900 MET-min/week, the decline in effect size became less pronounced and gradually approached a plateau. However, when the exercise dose exceeded 1,000 MET-min/week, the effect size showed an upward trend, indicating that further increases in dosage were not associated with additional improvement in depressive symptoms. Notably, in the high-dose range, the 95% confidence intervals became substantially wider, suggesting greater uncertainty in the estimates within this interval. This widening of the confidence intervals indicates that the fitted curve was less stable in the higher-dose range than in the low-to-moderate dose range. Therefore, the upward trend observed beyond 1,000 MET-min/week should be interpreted cautiously, as this portion of the curve was supported by fewer study estimates and may be more sensitive to the distribution of available data.

### Subgroup analyses

#### Effects of exercise modality on post-stroke depression

**Figure 5:**
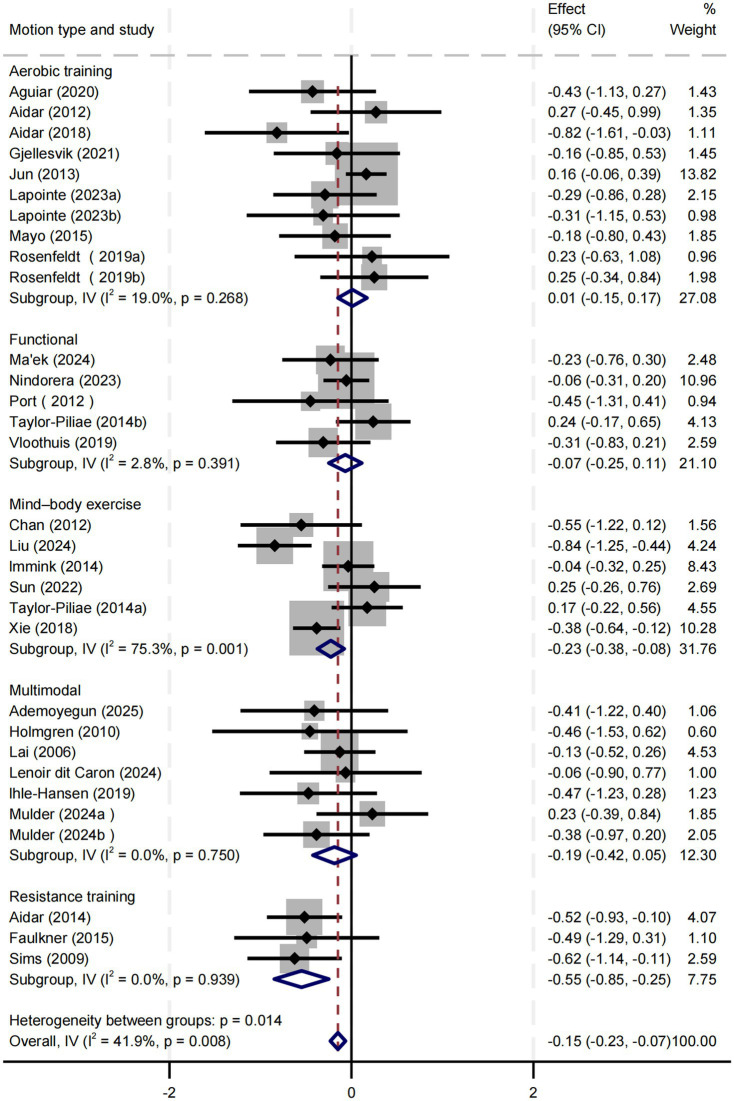
Subgroup analysis of exercise modality on post-stroke depression.

Subgroup analyses were conducted according to exercise modality, with studies categorized as aerobic training, functional training, mind–body exercise, multimodal training, and resistance training (Figure 5). Among these, the resistance training subgroup [SMD = −0.55, 95% CI (−0.85, −0.25)] and the mind–body exercise subgroup [SMD = −0.23, 95% CI (−0.38, −0.08)] demonstrated statistically significant effects. In contrast, aerobic exercise [SMD = 0.01], functional training [SMD = −0.07], and multimodal training [SMD = −0.19] did not reach statistical significance.

#### Effects of intervention duration on post-stroke depression

**Figure 6:**
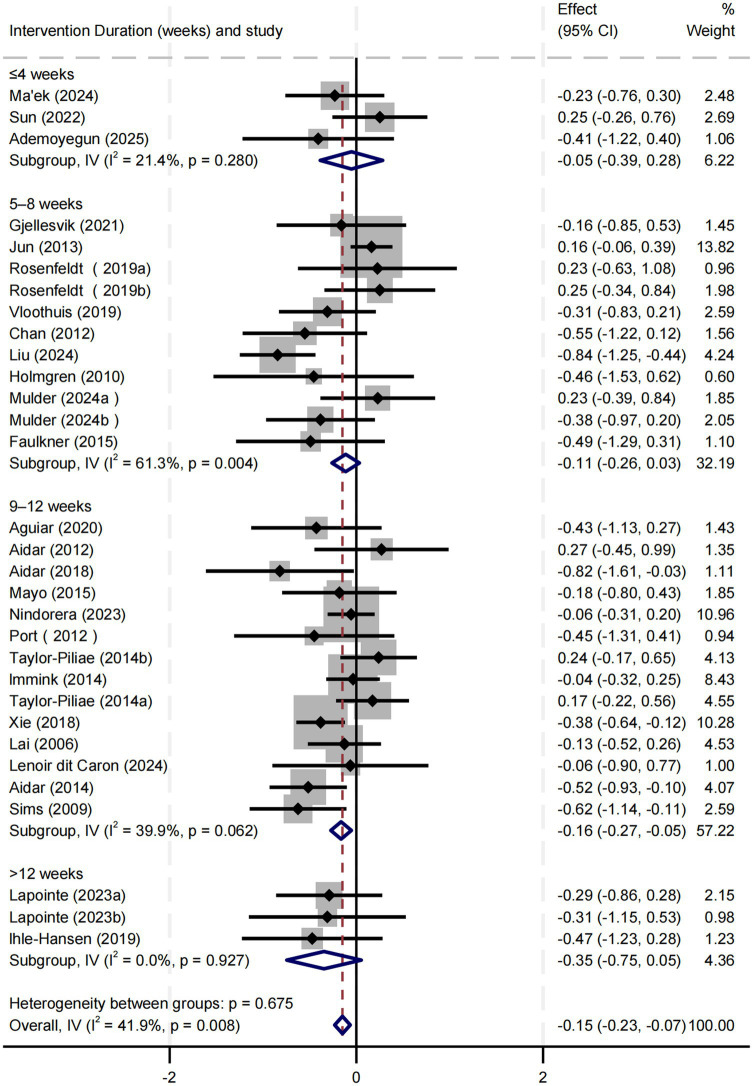
Subgroup analysis of intervention duration on post-stroke depression.

Subgroup analyses were also performed according to intervention duration, categorized as ≤4 weeks, 5–8 weeks, 9–12 weeks, and >12 weeks (Figure 6). A statistically significant pooled effect was observed only in the 9–12 weeks subgroup [SMD = −0.16, 95% CI (−0.27, −0.05)]. By contrast, the ≤4 weeks [SMD = −0.05], 5–8 weeks [SMD = −0.11], and >12 weeks [SMD = −0.35] subgroups did not show statistically significant effects.

#### Effects of session duration on post-stroke depression

**Figure 7:**
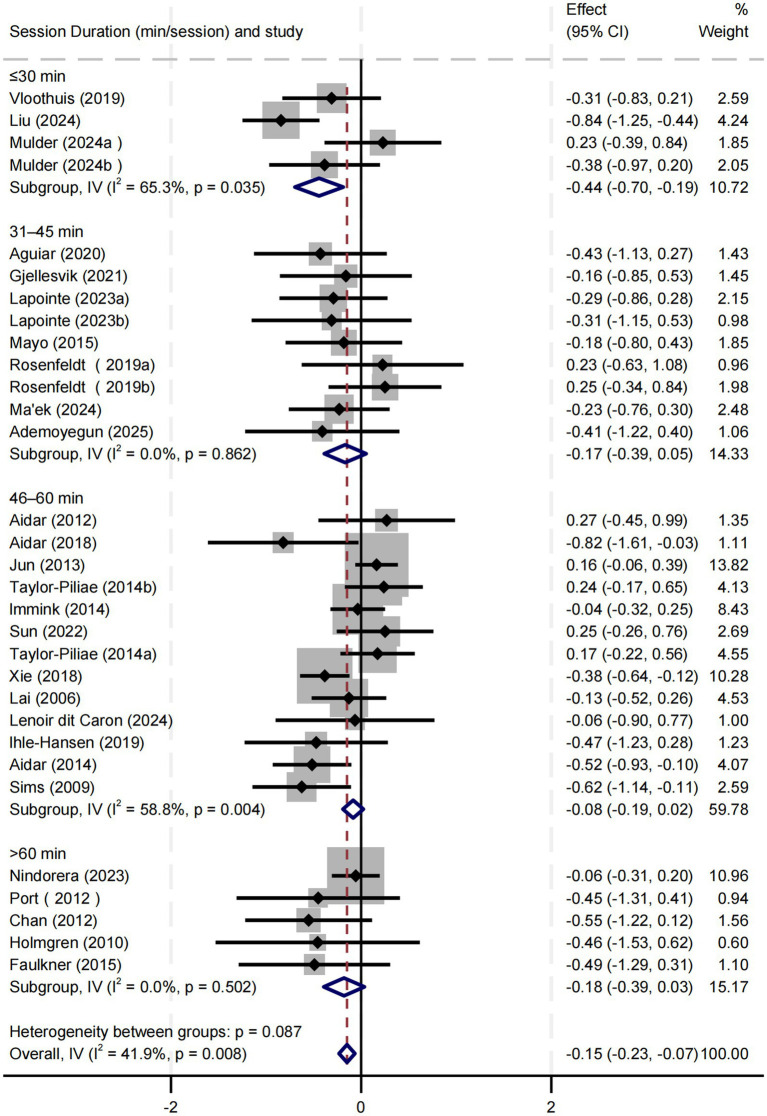
Subgroup analysis of session duration on post-stroke depression.

Subgroup analyses were performed according to session duration and categorized as ≤30 min, 31–45 min, 46–60 min, and >60 min (Figure 7). A statistically significant pooled effect was observed in the ≤30 min subgroup [SMD = −0.44, 95% CI (−0.70, −0.19)]. The remaining subgroups did not reach statistical significance, with corresponding effect sizes of 31–45 min [SMD = −0.17], 46–60 min [SMD = −0.08], and >60 min [SMD = −0.18].

#### Effects of exercise frequency on post-stroke depression

**Figure 8:**
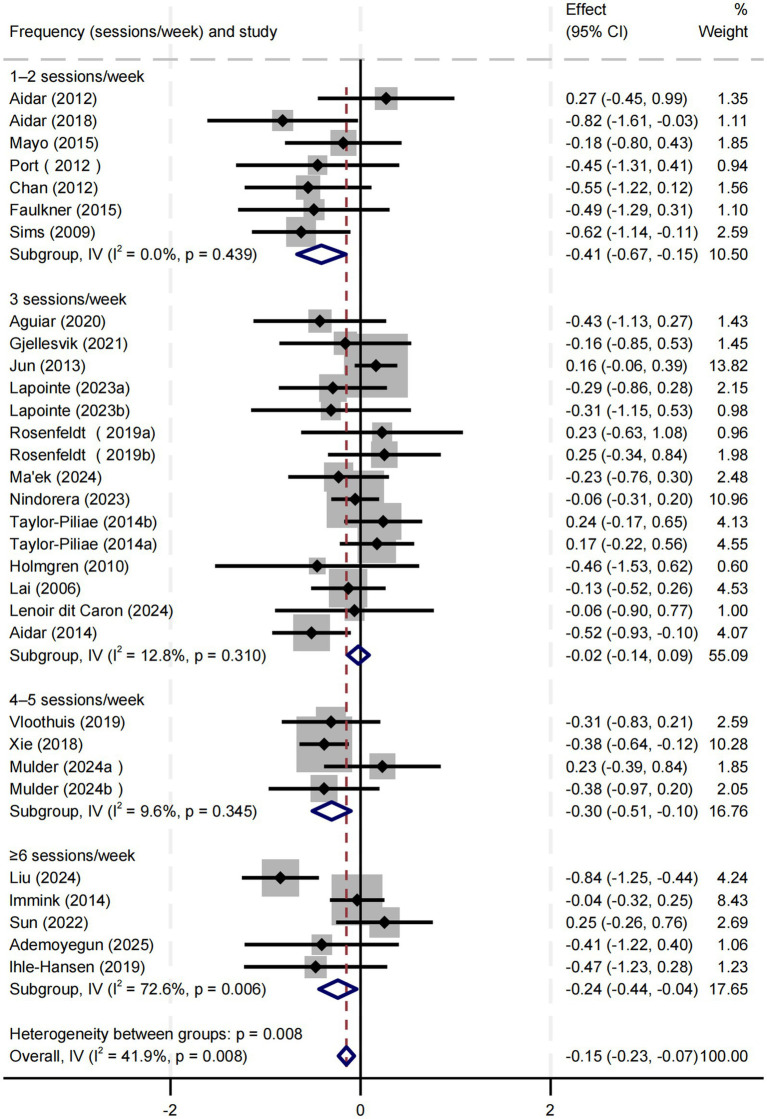
Subgroup analysis of exercise frequency on post-stroke depression.

Subgroup analyses were also conducted according to weekly exercise frequency, categorized as 1–2 sessions/week, 3 sessions/week, 4–5 sessions/week, and ≥6 sessions/week (Figure 8). Statistically significant pooled effects were observed in the 1–2 sessions/week subgroup [SMD = −0.41, 95% CI (−0.67, −0.15)], the 4–5 sessions/week subgroup [SMD = −0.30, 95% CI (−0.51, −0.10)], and the ≥6 sessions/week subgroup [SMD = −0.24, 95% CI (−0.44, −0.04)]. In contrast, the pooled effect in the 3 sessions/week subgroup [SMD = −0.02] did not reach statistical significance.

### Sensitivity analysis

A leave-one-out sensitivity analysis was conducted to evaluate the robustness of the pooled estimates (Supplementary material C). Sequential omission of individual studies did not materially alter the overall effect size; the re-estimated pooled effects consistently remained within the confidence interval of the original meta-analysis, approximately −0.33 to −0.03. No single study exerted a disproportionate influence on the summary results. These findings support the stability and reliability of the pooled effect estimates.

## Discussion

### Synthesis of evidence

The present study systematically evaluated the effectiveness of exercise interventions in improving PSD and applied a quantitative framework based on MET-min/week to explore the potential dose–response relationship between exercise dosage and depressive outcomes. By modeling the non-linear relationship between exercise dosage and treatment effects, we sought to provide an evidence-based reference for the development of individualized exercise prescriptions.

The findings of the present study indicate that exercise intervention was associated with an alleviation of depressive symptoms in patients with PSD, with a pooled effect size of [SMD = −0.15, 95% CI (−0.23, −0.07)] and moderate heterogeneity (*I*^2^ = 41.9%). Although between-study heterogeneity may have arisen from variations in intervention protocols and participant characteristics, the direction of effect was broadly consistent across trials, supporting exercise as a feasible non-pharmacological strategy for the management of PSD. Although the pooled effect size was relatively small, its clinical relevance should be interpreted within the broader context of post-stroke rehabilitation. Post-stroke depression is a multifactorial condition influenced by neurological recovery, physical disability, and psychosocial factors; therefore, large effects from a single non-pharmacological intervention may be difficult to achieve (51). Exercise may provide benefits beyond depressive symptom reduction, including improvements in physical function, mobility, and quality of life, which are central goals of stroke rehabilitation (52). From a public health perspective, even modest effects may have meaningful value when applied across a large population of stroke survivors. Nevertheless, the clinical significance of the observed effect should be further evaluated in future studies.

Further dose–response modeling suggested that the association between exercise dosage and improvement in post-stroke depressive symptoms was not linearly incremental. It is also important to interpret this pattern in light of the distribution of evidence across the dose range. The curve was more informative in the low-to-moderate dose range, where the confidence intervals were relatively narrower. By contrast, beyond approximately 1,000 MET-min/week, the confidence intervals widened, indicating lower precision of the estimated effects in this part of the curve. This does not contradict the observed non-linear association, but it suggests that the right-hand portion of the curve should be interpreted more cautiously. From an exercise intervention perspective, this is clinically plausible because stroke survivors often experience fatigue, reduced exercise tolerance, and fluctuating rehabilitation participation; therefore, higher prescribed doses may not always translate into greater completed exercise exposure or stronger mood-related benefits. The therapeutic effect reached its predicted maximum at approximately 801 MET-min/week, corresponding to the nadir of Hedges’ g. Beyond this threshold, further increases in exercise dosage were associated with a plateau in benefit or even an attenuation of effect. To assess the robustness of the findings, an additional random-effects model analysis was performed. The pooled effect estimate remained statistically significant (SMD = −0.18, 95% CI: −0.31 to −0.06) and was consistent with the primary fixed-effect model result (SMD = −0.15, 95% CI: −0.23 to −0.07). These findings suggest that the overall conclusions were not materially influenced by the choice of statistical model. Furthermore, the relatively wide prediction interval suggests that the magnitude of treatment effects may vary across future clinical settings. This finding is consistent with the moderate heterogeneity observed among the included studies. Nevertheless, both the fixed-effect and random-effects models yielded statistically significant pooled estimates, supporting the overall beneficial association between exercise intervention and improvements in post-stroke depressive symptoms.

Notably, the World Health Organization recommends that adults engage in at least 150–300 min of moderate-intensity or 75–150 min of vigorous-intensity physical activity per week (53), equivalent to an estimated 600–1,200 MET-min/week. The dose peak identified in the present study, approximately 801 MET-min/week, falls within the lower-to-mid range of these recommendations. This finding suggests that, among patients with post-stroke depression, an exercise dose within an appropriate range may be associated with stable and clinically meaningful improvements in mood. From a clinical perspective, this dosage range may offer advantages in terms of safety, feasibility, and adherence. These observations are consistent with previous evidence indicating that structured moderate-intensity exercise interventions can produce observable reductions in depressive symptoms over short- to mid-term intervention periods in individuals with stroke or other neurological disorders (54–56), whereas high-intensity protocols do not appear to yield proportionally greater benefits and may be more prone to fatigue accumulation and reduced adherence (57). Similarly, in older adults with depression, individuals with mild cognitive impairment, and populations with autism spectrum disorder, moderate-intensity exercise has demonstrated relative advantages in safety, participation, and consistency of effect (58, 59).

In contrast, only a limited number of studies have reported a positive association between high-intensity physical activity and mood improvement in specific populations (60, 61). Such discrepancies may reflect differences in functional capacity, baseline severity of depression, and study design. For example, cross-sectional studies are more likely to capture associations between overall physical activity levels and mental health status (62). By contrast, the present analysis was based on RCTs of structured interventions, which are better suited to delineating a clinically actionable optimal dosage range under controlled conditions while minimizing the influence of confounding factors.

Subgroup analyses further clarified the observed dose–response pattern at the level of specific prescription components. When stratified by exercise modality, only the resistance training subgroup demonstrated a statistically significant pooled effect [SMD = −0.55, 95% CI (−0.85, −0.25)]. Mind–body exercise may improve depressive symptoms through mechanisms beyond those associated with physical activity alone. By integrating movement, breathing regulation, and attentional control, mind–body exercise may enhance autonomic nervous system regulation, reduce stress-related HPA axis activation, and improve emotional regulation. In addition, the mindfulness-related components of these interventions may help reduce psychological distress and maladaptive rumination, thereby contributing to improvements in depressive symptoms (63). Given their relatively low physical demands, mind–body exercises may also be particularly suitable for stroke survivors and may promote better adherence to rehabilitation programs (64). One possible explanation is that, in addition to influencing depressive symptoms through biological pathways shared by exercise more broadly, such as reducing inflammatory responses, modulating neurotransmitter function, and promoting neuroplasticity, resistance training may also more directly target the muscle weakness and functional limitations commonly observed after stroke. Previous evidence suggests that resistance training can improve muscle strength in stroke survivors and, to some extent, reduce functional limitations while enhancing independence and participation in daily activities. These gains in recovery may in turn help alleviate feelings of helplessness and strengthen confidence in rehabilitation, thereby indirectly improving depressive symptoms. Moreover, because resistance training is typically goal-oriented and provides direct feedback, it may confer additional psychological benefits by enhancing self-efficacy and motivation for rehabilitation. An alternative explanation may relate to exercise intensity. Given that lower-frequency and shorter-duration interventions were associated with improvements in depressive symptoms, achieving a comparable weekly exercise dose may require relatively higher exercise intensity per session (65). Resistance training is often characterized by greater metabolic demands than some forms of aerobic exercise, and exercise intensity may therefore partly contribute to the observed differences between exercise modalities (66). However, because exercise intensity was not consistently reported across the included studies, this hypothesis could not be formally examined and warrants further investigation.

A statistically significant pooled effect was observed only in the 9–12 weeks subgroup [SMD = −0.16], whereas the ≤4 weeks, 5–8 weeks, and >12 weeks subgroups did not demonstrate statistical significance. Notably, substantial heterogeneity was detected in the 5–8 weeks subgroup (*I*^2^ = 61.3%). These findings suggest that achieving stable mood-related benefits in PSD may require a minimum cumulative exposure period; however, this should not be interpreted as indicating a simple linear increase in benefit over time. Although the >12 weeks subgroup showed a larger estimated effect size [SMD = −0.35], the result did not reach statistical significance, possibly because of limited sample size. Accordingly, the long-term effects of exercise intervention still require further verification.

Subgroup analysis by session duration further showed that only the ≤30 min subgroup demonstrated a statistically significant pooled effect [SMD = −0.44, 95% CI (−0.70, −0.19)]. One possible explanation is that stroke survivors often experience reduced physical capacity and heightened subjective fatigue, such that longer training sessions may impose greater physiological and psychological burden, thereby adversely affecting the exercise experience and sustained participation. By contrast, shorter sessions are generally more tolerable and more consistent with the principle of gradual progression in rehabilitation, which may help preserve adherence and support the accumulation of stable psychological benefits.

Regarding exercise frequency, the test for subgroup differences reached statistical significance (*p* = 0.031). Significant pooled effects were observed in the 1–2 sessions/week, 4–5 sessions/week, and ≥6 sessions/week subgroups, with SMDs of −0.41, −0.30, and −0.24, respectively, whereas the 3 sessions/week subgroup did not demonstrate statistical significance. Notably, substantial heterogeneity was present in the ≥6 sessions/week subgroup (*I*^2^ = 72.6%). This frequency pattern is directionally consistent with the overall U-shaped dose–response curve: insufficient frequency may fail to provide a sustained therapeutic stimulus, whereas excessively high frequency may attenuate mood-related benefits because of cumulative fatigue, inadequate recovery, or reduced adherence.

The present findings suggest that, in clinical practice for individuals with PSD, a structured exercise program delivered at a moderate weekly dose, approximately 801 MET-min/week, may offer relative advantages. More favorable improvements were more commonly observed in interventions characterized by resistance training [SMD = −0.55, 95% CI (−0.85, −0.25)], a frequency of 1–2 sessions/week [SMD = −0.41, 95% CI (−0.67, −0.15)], a session duration of ≤30 min [SMD = −0.44, 95% CI (−0.70, −0.19)], and an intervention duration of 9–12 weeks [SMD = −0.16, 95% CI (−0.27, −0.05)]. This combination of prescription characteristics may facilitate meaningful improvement in PSD while balancing safety, feasibility, and adherence, thereby avoiding excessive reliance on overly high training loads. As such, this evidence-informed framework provides a practical reference for individualized exercise prescription in PSD and may enhance both the stability of intervention effects and patient adherence (52). Nevertheless, extending this dosage paradigm to depression associated with other neurological disorders will require further investigation through rigorously designed trials.

### Clinical implications of the dose–response findings

The present analysis supports the role of exercise as a potentially valuable non-pharmacological strategy for improving emotional and psychological well-being among patients with post-stroke depression. At the study level, moderate-dose exercise interventions were associated with greater attenuation of depressive symptoms, which may reflect a more favorable balance between therapeutic stimulation and intervention feasibility. This interpretation is clinically plausible, as stroke survivors often experience functional limitations, reduced exercise tolerance, and fatigue, all of which may hinder sustained participation in high-intensity or high-volume training programs (67, 68). Therefore, rather than implying a fixed individual-level dose threshold, the present findings suggest that moderate, progressively structured exercise programs may represent a practical direction for future intervention design and rehabilitation research. This is also consistent with previous evidence showing that moderate-intensity, structured, and sustainable exercise programs can produce relatively stable antidepressant effects in populations with stroke and other neurological disorders (54, 55).

These findings further highlight the importance of individualized exercise prescription. Intervention design should take into account patients’ physical capacity, severity of depressive symptoms, comorbidities, and environmental context, in line with the principles of patient-centered rehabilitation (69). Although the present study confirmed the overall benefits of exercise intervention, the exercise dose used in this analysis was derived from prescribed intervention parameters rather than the actual completed dose. In clinical settings, discrepancies often exist between prescribed and performed exercise because of differences in adherence, physical tolerance, and treatment-related adverse effects. Such discrepancies may lead to exposure misclassification and potentially bias the estimation of the dose–response relationship. Therefore, the findings of this study should still be interpreted with caution when applied to clinical practice.

### Strengths and limitations

To the best of our knowledge, this is the first dose–response meta-analysis to systematically evaluate the effects of exercise interventions on post-stroke depression (PSD), and it used a quantitative framework based on metabolic equivalent minutes per week (MET-min/week) to standardize exercise dosage across studies. This review was grounded in a comprehensive search of 5 major databases and employed rigorous methodological procedures, including independent duplicate screening, subgroup analyses, and sensitivity analyses, thereby enhancing the robustness and credibility of the findings. The present results provide a useful reference for optimizing exercise dosage and developing individualized exercise prescriptions for PSD. Nevertheless, several limitations should be acknowledged.

First, only English-language publications were included, which may have introduced language bias and resulted in the omission of potentially relevant RCTs, particularly studies of mind–body exercise, such as Tai Chi and Qigong, which are more common in non-English-speaking regions and are often published in local journals. Although the included studies were conducted in China, the United States, Canada, Brazil, Australia, and several European countries, the overall evidence base remains derived predominantly from high-income countries, with limited representation from low-resource settings. Second, differences in rehabilitation systems, healthcare resources, and the cultural acceptability of exercise across regions may restrict the generalizability of the findings. Furthermore, adherence to exercise interventions was insufficiently reported in many studies, and the prescribed exercise dose may not have corresponded to the dose actually completed by participants, potentially leading to exposure misclassification and affecting the estimation of the optimal dose. Third, several subgroup analyses were based on a limited number of studies, which may have reduced statistical power. In addition, the duration of intervention in most RCTs was concentrated within 18 weeks, and evidence regarding long-term exercise interventions remains limited. Therefore, the long-term sustainability of exercise effects and the optimal maintenance dose require further investigation. Another limitation is that studies involving participants with severe psychiatric or neurological comorbidities were excluded, which may affect the generalizability of the findings. Further research is needed in these populations. Because the dose–response analysis was based on prescribed exercise doses rather than actual completed doses, and adherence data were inconsistently reported across studies, we could not evaluate the influence of completed exercise exposure on depressive outcomes; this issue may have been more pronounced in the high-dose range, where fewer studies contributed data and the confidence intervals became wider above approximately 1,000 MET-min/week.

Future research should incorporate multilingual evidence, improve the standardized reporting of exercise prescriptions and adherence, and conduct large-scale trials with longer intervention durations and extended follow-up periods. In light of these limitations, the findings of the present study should be interpreted with caution.

## Conclusion

The present findings suggest that exercise intervention may be associated with a modest improvement in depressive symptoms among patients with PSD. Dose–response analysis indicated a non-linear association between exercise dosage and symptom improvement, with a moderate dose, approximately 801 MET-min/week, approaching the model-predicted peak effect. Under the current evidence base, further increases in exercise dosage have not been associated with clear additional benefit.

However, the available evidence remains subject to several limitations. The restriction to English-language publications may have introduced language bias, and inadequate reporting of exercise adherence in some studies may have resulted in discrepancies between prescribed and actual completed exercise doses, thereby influencing estimation of the dose–response relationship. In addition, most included studies involved relatively short intervention periods, and robust evidence regarding long-term effects is still lacking.

Taken together, the current findings provide a preliminary reference for the individualized optimization of exercise prescriptions, but further high-quality RCTs with longer follow-up are still needed to validate and refine these conclusions.

## Data Availability

The original contributions presented in the study are included in the article/supplementary material, further inquiries can be directed to the corresponding author.
