# Supplementary material for: Nonlinear dose–response effects of exercise interventions on post-stroke depression: a systematic review and meta-analysis
Source: Front Public Health. 2026 Jul 14;14:1876753. doi: 10.3389/fpubh.2026.1876753 (PMC13407525; doi:10.3389/fpubh.2026.1876753)
Supplement: Supplementary file 1 [file Supplementary_file_1.docx]

Section A

| **Detailed Search Strategies for Each Database** | |
| --- | --- |
| PubMed search: April 16, 2026 |  |
| (("Stroke"[Mesh] OR "Cerebrovascular Accident"[Mesh] OR "Stroke Patients" OR "Cerebrovascular Accident Patients" OR "stroke" OR "CVA" OR "ischemic stroke" OR "hemorrhagic stroke" OR "stroke rehabilitation" OR "post-stroke" OR "stroke recovery" OR "stroke sequelae" OR "stroke survivors" OR "brain injury" OR "neurovascular disorder")  AND  ("Exercise"[Mesh] OR "Exercise Therapy"[Mesh] OR "Motor Activity"[Mesh] OR "Physical Fitness"[Mesh] OR "aerobic training" OR "resistance training" OR "fitness program" OR "neurological rehabilitation" OR "exercise intervention" OR "rehabilitation exercise" OR "post-stroke rehabilitation" OR "strength training" OR "high-intensity interval training" OR "functional exercise" OR "circuit training" OR "balance training")  AND  ("Depression"[Mesh] OR "Depressive Disorder"[Mesh] OR "Depression in Stroke" OR "post-stroke depression" OR "depressive disorders" OR "anxiety" OR "mood disorders" OR "cognitive dysfunction" OR "neuropsychiatric symptoms" OR "psychological well-being" OR "fatigue" OR "emotional disturbances")  AND  ("Child"[Mesh] OR "Adolescent"[Mesh] OR "Pediatrics"[Mesh] OR "Adult"[Mesh] OR "Aged"[Mesh] OR "Elderly" OR "middle-aged adults" OR "young adults" OR "older adults" OR "elderly stroke patients")) | 818 |
| Web of Science search: April 16, 2026 |  |
| TS=("stroke" OR "ischemic stroke" OR "hemorrhagic stroke" OR "post-stroke" OR "stroke rehabilitation" OR "brain injury") AND TS=("exercise" OR "exercise therapy" OR "physical activity" OR "aerobic training" OR "rehabilitation") AND TS=("depression" OR "post-stroke depression" OR "depressive symptoms" OR "mental health") AND TS=("intensity" OR "duration" OR "exercise prescription") | 714 |
| Cochrane Library search: April 16, 2026 |  |
| #1  "stroke":ti,ab,kw OR "cerebrovascular accident":ti,ab,kw OR "CVA":ti,ab,kw OR "brain infarction":ti,ab,kw OR "ischemic stroke":ti,ab,kw OR "hemorrhagic stroke":ti,ab,kw OR "post-stroke":ti,ab,kw OR "stroke survivor" NEXT *:ti,ab,kw OR "stroke patient" NEXT *:ti,ab,kw OR "stroke rehabilitation":ti,ab,kw  #2  "exercise":ti,ab,kw OR "physical activity":ti,ab,kw OR "aerobic exercise":ti,ab,kw OR "resistance training":ti,ab,kw OR "strength training":ti,ab,kw OR "exercise therapy":ti,ab,kw OR "exercise intervention" NEXT *:ti,ab,kw OR "exercise program" NEXT *:ti,ab,kw OR "physical exercise":ti,ab,kw OR "rehabilitation exercise":ti,ab,kw OR "therapeutic exercise":ti,ab,kw OR "motor activity":ti,ab,kw OR "training intervention":ti,ab,kw OR "exercise prescription":ti,ab,kw OR "walking program":ti,ab,kw OR "cycling":ti,ab,kw OR "treadmill training":ti,ab,kw  #3 "depression":ti,ab,kw OR "depressive disorder" NEXT *:ti,ab,kw OR "depressive symptom" NEXT *:ti,ab,kw OR "mood disorder" NEXT *:ti,ab,kw OR "psychological distress":ti,ab,kw OR "emotional disorder":ti,ab,kw OR "post-stroke depression":ti,ab,kw OR "PSD":ti,ab,kw OR "depressed mood":ti,ab,kw OR "mental health":ti,ab,kw OR "affective symptom" NEXT *:ti,ab,kw  #1 AND #2 AND #3 | 601 |
| Embase search: April 16, 2026 |  |
| ('stroke':ti,ab,kw OR 'accident, cerebrovascular':ti,ab,kw OR 'acute cerebrovascular lesion':ti,ab,kw OR 'acute focal cerebral vasculopathy':ti,ab,kw OR 'acute stroke':ti,ab,kw OR 'apoplectic stroke':ti,ab,kw OR apoplexia:ti,ab,kw OR apoplexy:ti,ab,kw OR 'blood flow disturbance, brain':ti,ab,kw) AND ('exercise':ti,ab,kw OR 'biometric exercise':ti,ab,kw OR effort:ti,ab,kw OR 'exercise capacity':ti,ab,kw OR 'exercise performance':ti,ab,kw OR 'exercise training':ti,ab,kw OR exertion:ti,ab,kw OR 'fitness training':ti,ab,kw OR 'physical conditioning, human':ti,ab,kw) AND ('depression':ti,ab,kw OR 'central depression':ti,ab,kw OR 'clinical depression':ti,ab,kw OR 'depressive disease':ti,ab,kw OR 'depressive disorder':ti,ab,kw OR 'depressive episode':ti,ab,kw OR 'depressive illness':ti,ab,kw OR 'depressive personality disorder':ti,ab,kw OR 'depressive state':ti,ab,kw) | 1078 |
| Scopus search: April 16, 2026 |  |
| TITLE-ABS-KEY("stroke" OR "ischemic stroke" OR "post-stroke" OR "cerebrovascular accident" OR "brain infarction" OR "hemorrhagic stroke" OR "stroke recovery" OR "brain injury")  AND  TITLE-ABS-KEY("exercise" OR "physical activity" OR "motor activity" OR "aerobic training" OR "resistance training" OR "movement therapy" OR "fitness program")  AND  TITLE-ABS-KEY("post-stroke depression" OR "stroke-related depression" OR "depressive disorder" OR "depressive symptoms" OR "mood disorder" OR "affective disorder" OR "clinical depression") | 901 |

Section B


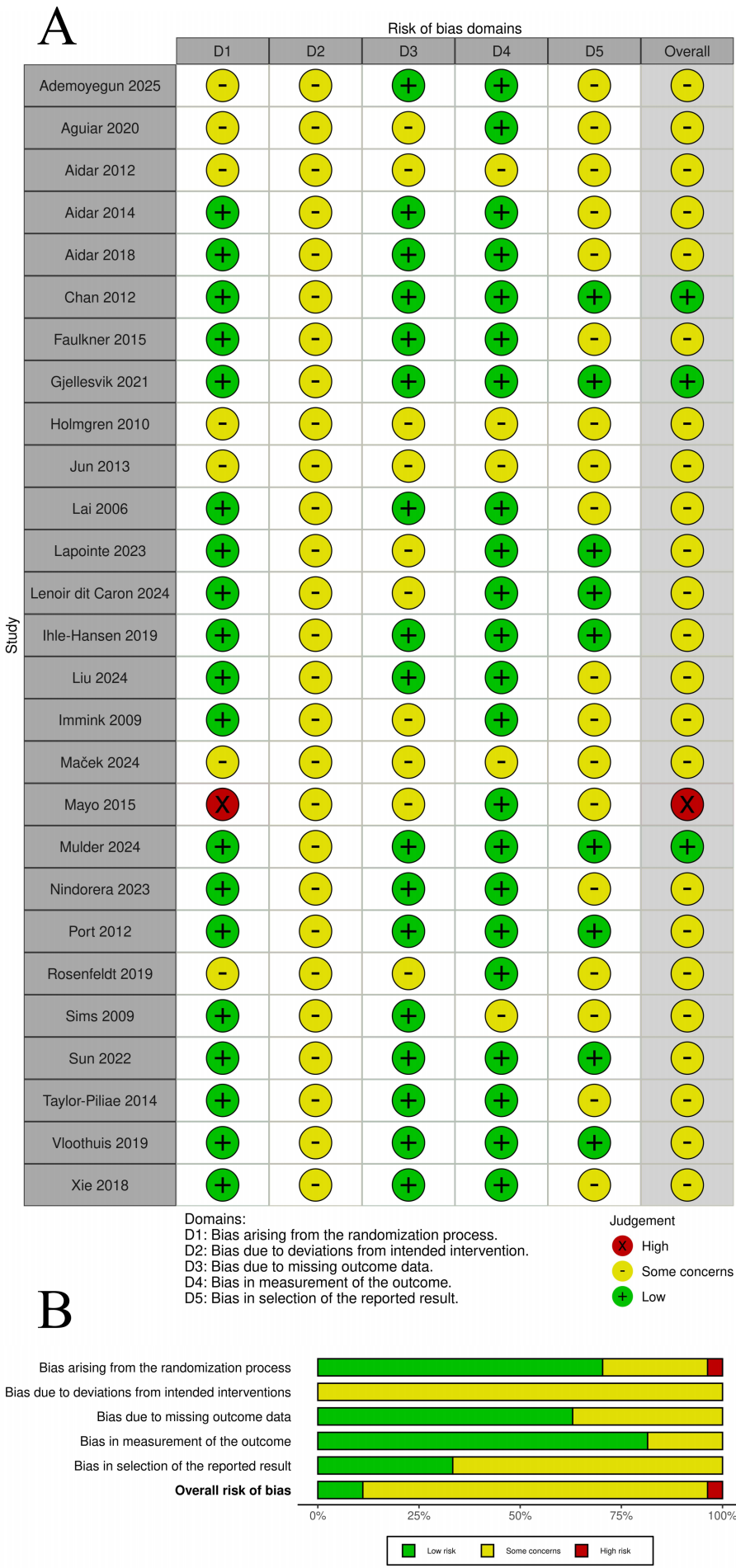


**Figure** (A) Distribution of methodological quality across domains; (B) Risk-of-bias graph and summary.

Section C


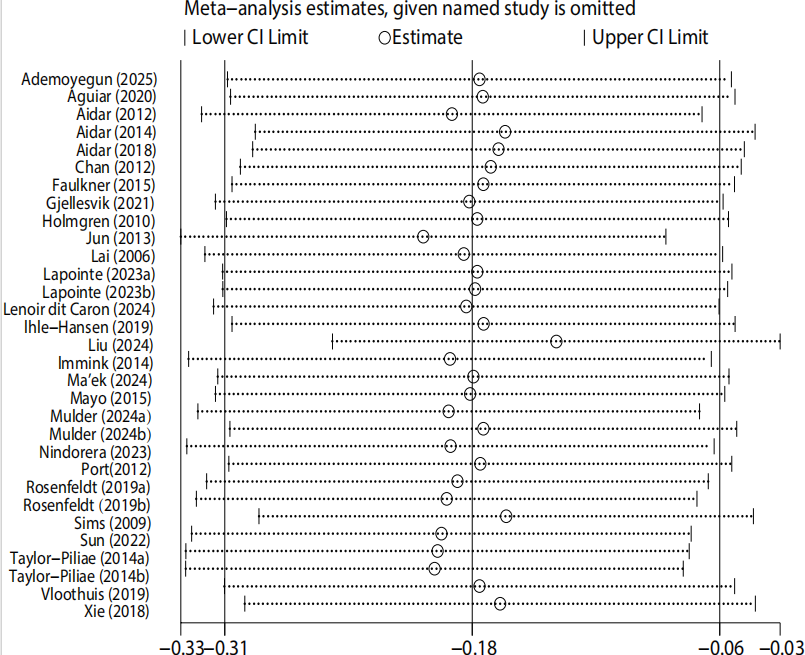


**Supplementary Figures 1**. Sensitivity analysis
